# Global Mental Health and Pharmacology: The Case of Attention Deficit and Hyperactivity Disorders in Brazil

**DOI:** 10.3389/fsoc.2020.535125

**Published:** 2020-09-29

**Authors:** Francisco Ortega, Manuela Rodrigues Müller

**Affiliations:** Rio de Janeiro State University, Rio de Janeiro, Brazil

**Keywords:** global mental health, attention deficit and hyperactivity disorder, medicalization, methylphenidate, treatment gap, child and adolescent mental health

## Abstract

Global Mental Health (GMH) is the field of study, research, and intervention, which aims at improving access to mental health worldwide. It is based on the global burden of disease research program and on the existence of a large “treatment gap” between the need and availability of mental health services, displaying individual and social costs of undiagnosed and untreated mental disorders, especially in low- and middle-income countries (LMIC). Few academic publications in Brazil dialogue directly with the field of GMH, although several issues drawn from its agenda have been the subject of mental health policies in the country. Brazil can be classified as a middle-income country with a well-structured national health system. This system is oriented toward primary health care, which integrates both community mental health services and the broader health care network. The debate between GMH advocates and critics has unearthed old controversies in psychiatry such as universality or cultural specificity of mental disorders, their expressions, and their relationship with social and economic factors. We intend to examine how these controversies reverberate in the Brazilian mental health scenario, taking as an illustration the debates around Attention Deficit Hyperactivity Disorder (ADHD) in the country. ADHD discussions oppose those who argue that the condition is underdiagnosed and undertreated, and those who claim that there is overdiagnosis and overtreatment and thus, medicalization of childhood. This article presents the current status of the Brazilian mental health literature on ADHD, with emphasis on tensions around diagnosis, prevalence and interventions. Our aim is to highlight how the differential in discourse shapes the debate on ADHD in Brazil and how this may contribute to the GMH agenda. This goal will be undertaken in three steps. First, we will briefly examine studies around GMH and ADHD. Secondly, we address Brazilian studies on this theme, considering the specificities regarding the constitution of the mental health field. Finally, we will examine the debate of treatment gap vs. medicalization in the country in order to underscore the potentials and limitations of each perspective.

## Introduction

Global Mental Health (GMH) is a field of study, research and practice, which aims at improving access and ensuring equity in mental health care for all people in the world (Patel and Prince, 2010). GMH researchers highlight the existence of a treatment gap in mental health around the globe and promote strategies to address the situation (Chisholm et al., 2007; Jacob et al., 2007; Patel et al., 2007; Prince et al., 2007; Saraceno et al., 2007; Saxena et al., 2007; Patel, 2012).

The GMH agenda has also raised important criticism especially from transcultural psychiatrists and anthropologists. Overall, such objections outline the biology vs. culture controversies and denounce the neglect of social, political, and economic processes associated to mental health diagnostics, treatments, and research (Summerfield, 2012; Clark, 2014; Kirmayer and Swartz, 2014; Ortega and Wenceslau, 2020).

Attention Deficit Hyperactivity Disorders (ADHD) are among the mental health conditions singled out by GMH research and interventions (Kieling et al., 2011; Patel et al., 2013, 2018; Ordóñez and Collins, 2015). The issue of accessibility to pharmacological treatments for ADHD raises important questions for GMH. Estimating prevalence for the condition is one of the aspects to be considered. Early detection and diagnosis are believed to be central for management of ADHD cases (Flisher et al., 2010). Researchers have examined the obstacles to achieve this goal in low- and middle-income countries (LMIC), such as limited resources and lack of mental health specialists, and have proposed that diagnosis and treatments may be achieved by raising awareness and empowering community health care teams, educators and school counselors along with other lay agents in the community (Flisher et al., 2010).

There are strong controversies between those who advocate that ADHD remains globally prevalent but undiagnosed and inadequately treated (Polanczyk et al., 2007; Flisher et al., 2010) and those who understand that it is a social construct, reflecting the advancement of both US psychiatry and the pharmaceutical industry (Singh et al., 2013; Conrad and Bergey, 2014; Mills, 2014; Conrad and Singh, 2018). GMH initiatives highlight the neurobiological nature of the disorder and promote the access to psychostimulants and other pharmacological agents in LMIC (Flisher et al., 2010). Assuming the universal, neurobiological nature of the condition that would affect equally all individuals across cultural contexts and nations justifies the scalability and generalizability of the use of psychostimulants as well as the exportation of Western psychiatric expertise and standardized care packages to the Global South (Mills, 2014). Critics argue that such strategies privilege one-size-fits all interventions, leaving aside difference markers such as gender, race, or culture. The result is the rapid expansion of diagnosis and pharmaceutical treatments–what has been called the “McDonaldization of children's health” (Timimi, 2010; Mills, 2014). Although we sympathize with those perspectives, they frequently hide deep differences and important negotiations in the way individual countries have engaged with ADHD. While some countries have rapidly accepted the US biomedical model and consequently have diagnosed and treated with psychostimulants large parts of their children and adult population, other countries, like Brazil, have challenged and rejected biomedical models (Smith, 2017).

Controversies around ADHD play in different ways across diverse national configurations and should be considered when promoting GMH strategies for the condition. Moreover, there are still few studies that focus on the provision of evidence-based care for this population in LMIC. Most investigations center around pharmaceutical treatment strategies originally implemented in high income countries (Patel et al., 2013, 2018; Ordóñez and Collins, 2015) and set aside local histories, perspectives and approaches (Smith, 2017).

The specifics of the different national contexts impact a global agenda for ADHD (Smith, 2017) and are seldom addressed from a social science perspective. It is not possible to make generalizations about the phenomenon without the risk of reducing the dynamics, manifestations and results associated with its dissemination (Conrad and Singh, 2018).

Thus, ADHD is an interesting case to examine social and structural processes that permeate the transmission of psychiatric diagnoses and treatments, as well as their differences across national contexts. Literature on the issue draws on two key-concepts from social sciences, medicalization and globalization (Singh et al., 2013; Conrad and Bergey, 2014; Smith, 2017; Conrad and Singh, 2018). In this sense, ADHD illustrates the overarching globalization of a medicalized category (Conrad and Singh, 2018).

This article examines how global issues and controversies in global (mental) health take specific forms within particular socioeconomic, political and historical contexts focusing on the debates surrounding ADHD in Brazil. The national history of ADHD in the country discloses the influence of social, political and cultural factors in the framing of diagnosis and treatments. The debates around the condition in Brazil are also distinguished by tensions between those who argue that the condition is underdiagnosed and undertreated, and those who claim that there is overdiagnosis and overtreatment and thus medicalization of childhood.

This article presents the current status of the Brazilian mental health literature on ADHD, with emphasis on tensions around diagnosis, prevalence and interventions, organization of care and health policies. Our aim is to highlight how different discourses shape the debate on ADHD in the country and how they can contribute to the GMH agenda. This goal will be accomplished in three steps. First, we will briefly examine studies around GMH and ADHD. Next, we will address the Brazilian studies on this subject, considering the specificities of the constitution of the mental health field. Finally, we will critically analyze the debate over treatment gap vs. medicalization in the country in order to examine potentials and limits of each perspective.

The contrast between these two perspectives regarding ADHD (treatment gap or medicalization) can display assumptions about mental disorders as universal biological conditions or as social constructs, which limit the dialogue and proposals for this population. GMH strategies highlight the notion of treatment gap as evidence of the existence of a portion of the world population living with mental distress and without access to health care (Jacob et al., 2007; Patel et al., 2007; Saxena et al., 2007). Children and adolescents are among the most affected, and ADHD is one of the conditions recognized by GMH to contribute to this gap. Although the care and inclusion of children and adolescents living with mental distress should have urgent attention, it is also essential to understand how this index (treatment gap) is used in specific contexts and what effects it may have.

Moreover, ADHD has been one of the main entities studied by medicalization scholars (Singh et al., 2013; Conrad and Bergey, 2014). In this sense, it is important to examine the different configurations involved in the expansion of the diagnosis globally and the role played by diverse movements, such as GMH, and the debates they raise (Clark, 2014; Conrad and Bergey, 2014).

## Global Mental Health Agenda for ADHD

### Childhood and Adolescence Mental Disorders and Global Mental Health

The objectives of Global Mental Health include producing knowledge about mental health from a global perspective, promoting research and evaluation of mental illness and care strategies, mitigating the treatment gap, ensuring access, and equity in mental health care and advocating for the rights of people with mental disorders and their families at a global level (Chisholm et al., 2007; Jacob et al., 2007; Patel et al., 2007; Prince et al., 2007; Saraceno et al., 2007; Saxena et al., 2007; Patel and Prince, 2010; Patel, 2012).

To achieve these goals, GMH supporters stress the role of community mental health services and primary care facilities, and favor evidence-based, collaborative interventions that are responsive to local characteristics and scalable to broaden the access to mental health care (Chisholm et al., 2007; Jacob et al., 2007; Patel et al., 2007; Prince et al., 2007; Saraceno et al., 2007; Saxena et al., 2007).

Since its emergence in 2007, GMH researchers have produced a significant amount of empirical studies focused on low- and middle-income countries (LMICs), especially those in sub-Saharan Africa and Southern Asia. Conversely, there is a tendency in these studies to prioritize various conditions, such as depression, psychotic, and stress-related disorders, but with limited attention to contextual and socio-demographic aspects (Misra et al., 2019).

GMH initiatives have revitalized old controversies in psychiatry around the universality or cultural specificity of mental disorders. Critics of GMH argue that it promotes a Western, biomedical model of illness and treatments as well as the expansion of the Pharma industry; neglects practitioners of traditional therapies; disregards cultural influences on cause, course and outcome of mental disorders, as well as explanations for mental distress; medicalizes suffering and ignores social and economic determinants of mental health (Summerfield, 2012; Clark, 2014; Kirmayer and Swartz, 2014; Ortega and Wenceslau, 2020). GMH advocates have refuted such criticism and stressed their engagement with local communities and attention to context and culture in the design of the interventions. Furthermore, they have also underlined the deep influence of social sciences and cultural psychiatry in their methods and principles and their strong concern for human rights (Patel, 2014).

Mental disorders in childhood and adolescence, especially ADHD, constitute an interesting case to examine controversies around GMH and how these are instantiated differently across the diverse national contexts. There is an important treatment gap for these disorders as well as those victims of abuse and neglect, particularly in LMICs, where 90% of the world's children and adolescents reside and represent 50% of the local population (Kessler et al., 2007; Collins et al., 2011; Gore et al., 2011; Kieling et al., 2011; Barry et al., 2013; Patel et al., 2013; Ordóñez and Collins, 2015). According to the literature, mental disorders account for the higher burden of disease in children and young people, including developmental disabilities (such as intellectual disability and autism), emotional disorders (mainly anxiety and depression), and disruptive behavioral disorders (ADHD and conduct disorders), with lasting lifelong impact in terms of health and employment options, as well as to health systems (Patel and Prince, 2010; Kieling et al., 2011; Patel et al., 2013; Ordóñez and Collins, 2015).

Strategies to address both treatment and research gaps in child and adolescent mental health are equivalent to those aimed at adults, for they involve the adoption of a perspective that incorporates life-course and system-wide approaches, as well as evidence-based initiatives (Collins et al., 2011; Kieling et al., 2011; Ordóñez and Collins, 2015; Orr and Bindi, 2017). GMH professionals propose complementary pathways to overcome these barriers. Early diagnosis, increased awareness of family members and education professionals, development of collaboration among schools, social care and the legal system, and skilled mental health professionals are believed to be key features for addressing mental disorders and granting individuals access to quality mental health care (Flisher et al., 2010; Kieling et al., 2011; Barry et al., 2013; Patel et al., 2013).

### Clinical and Epidemiological Features of ADHD

ADHD is classified as a neurodevelopmental disorder within psychiatric and GMH literature (Flisher et al., 2010; American Psychiatric Association, 2014; Insel, 2014; Ordóñez and Collins, 2015). Brain developmental studies have contributed to this view of the condition (Insel, 2014). Evidence from high-income countries (HICs) suggests that ADHD is a syndrome with complex genetic etiological factors, in which 80% of cases may be related to genetic inheritance (Flisher et al., 2010). Social determinants however, would also influence symptomatology. Some identifiable factors are low socioeconomic status, low education, parental mental disorder, family conflicts and severe early deprivation (Flisher et al., 2010). Other non-genetic factors associated with ADHD are those that affect early brain development, such as perinatal distress, smoking, and alcohol use during pregnancy, low birth weight, obstetric complications, epilepsy, and HIV (Flisher et al., 2010). Although these findings corroborate the hypothesis that ADHD is a heterogeneous disorder influenced by interactions between genes and environment, there are comparatively fewer studies that delve into the interactions between genetics and environment in LMIC and as such, their findings do not always coincide with those observed in HICs (Flisher et al., 2010).

The global prevalence of ADHD is estimated to be between 5 and 7% (Polanczyk et al., 2007; Shaw et al., 2012; Raman et al., 2018), with ~2.5% prevalence in adults. ADHD may persist in up to 65% of adults diagnosed during childhood (Fayyad et al., 2007; Shaw et al., 2012; Raman et al., 2018). Persistence of the condition would be related to the severity of ADHD symptoms, psychosocial adversity, presence of psychiatric comorbidities, and occurrence of ADHD in relatives. It is common for adults with ADHD to go undiagnosed and therefore untreated (Shaw et al., 2012).

ADHD symptoms can often lead to functional impairment in many spheres, with repercussions on quality of life and increased risk of lifelong psychiatric comorbidities (Flisher et al., 2010; Shaw et al., 2012). These findings have led to a shift in the intervention focus. It is understood that childhood and adolescence constitute an opportune moment for health promotion, the improvement of social and emotional skills and cognitive development (Kieling et al., 2011; Kieling and Belfer, 2012; Barry et al., 2013; Insel, 2014; Ordóñez and Collins, 2015). Thus, in addition to improving the immediate symptoms of ADHD, long-term functionality has been sought (Flisher et al., 2010; Shaw et al., 2012).

### Global Mental Health Interventions for ADHD

GMH's approaches to ADHD stress the significant burden of disease for children and adolescents and life-long consequences associated with the condition. They also evince diverse diagnosis and treatment practices due to the fragility of health systems, shortage of trained professionals and stigma especially in LMIC. The need to address these problems leads to different proposals including sensitization of the population to risk factors, increasing recognition, and early detection in community settings such as schools, social welfare and the legal system, and the introduction of collaborative care (task-shifting) in contexts with shortage of skilled mental health professionals (Flisher et al., 2010; Kieling and Belfer, 2012; Barry et al., 2013; Patel et al., 2018). Moreover, the development of advocacy groups including professionals and individuals living with ADHD are also encouraged, especially in contexts of insufficient mental health professionals and community services (Flisher et al., 2010).

In general, the intervention with the strongest evidence involves the use of medication (Flisher et al., 2010; Patel et al., 2013; Raman et al., 2018). The preferred medications are psychostimulants (methylphenidate and amphetamine), given the findings on its efficacy and safety of usage (Flisher et al., 2010). Other interventions–e.g., psychotherapy, social skills training, psychoeducational interventions with caregivers–may be implemented in conjunction with psychopharmacological interventions (multimodal treatment) or without medication (Flisher et al., 2010; Kieling et al., 2011; Shaw et al., 2012; Patel et al., 2013; Beau-Lejdstrom et al., 2016; World Health Organization, 2016; National Institute for Health and Care Excellence (NICE), 2019). Drug prescription should be avoided whenever symptoms are mild to moderate, if there is diagnostic uncertainty and/or minimal functional impairment, and if there is a shortage of professionals and adequate services to attend to patients and their families (Flisher et al., 2010; Patel et al., 2018; National Institute for Health and Care Excellence (NICE), 2019).

Flisher et al. (2010) argue that the insufficiency of mental health professionals in LMIC should not be taken as an impediment to the introduction of packages of care in those countries. Those interventions would involve the screening of high-risk groups, psychoeducational interventions with caregivers, medication prescription and behavioral interventions.

Scholars agree that further studies are needed to assess the efficacy of ADHD interventions in LMICs (Flisher et al., 2010; Kieling et al., 2011; Patel et al., 2013). Patel et al. (2013) draw attention to the fact that, in addition to the scarcity of investigations regarding the treatment of ADHD in LMIC, the greater number of the existing studies focus on pharmacological strategies. Although studies aimed at evaluating multimodal interventions are increasing and providing meaningful results, the lack of evidence in relation to the effectiveness of psychosocial or combined interventions (“care packages”) is a key hurdle to scaling up care packages, as these demand significant contextual adaptation (Patel et al., 2013; Raman et al., 2018).

More recently, research has addressed issues on the combination of treatments, as well as the duration of effects of different treatments and their combinations (Galera et al., 2014; Arnold et al., 2015; Patel et al., 2018; Raman et al., 2018; Lam et al., 2019). The best results in multimodal treatments and their lasting effects may apparently be related to effects on neuroplasticity, as well as to coping strategies developed during the use of medication (Lam et al., 2019). Although there appear to be long-term benefits in multimodal approaches (Arnold et al., 2015; Lam et al., 2019), the use of different treatment response criteria and the preference for psychotherapeutic interventions based on cognitive behavioral therapy may not coincide with ongoing therapeutic experiences in LMICs. In Brazil, psychodynamic interventions (especially psychoanalysis) are largely used within community mental health services (Menezes et al., 2018).

Moreover, as noted by Galera et al. (2014), elements related to the sociocultural and economic contexts, such as parental educational level, parenting style, social integration (e.g., history of immigration or low socioeconomic status) are associated with iniquities in access to different therapeutic modalities, as well as to the continuity of their use. Individual characteristics are not the sole variables that influence drug exposure and those additional factors have to be considered in order to expand care alternatives and reduce the risk of stigmatization of users and their families.

A recent study by Raman et al. (2018) examined prevalence and tendencies in the use of medication for ADHD among children, adolescents and adults in the period from 2001 to 2015 in four regions: Asia and Australia (Hong Kong, Japan, Taiwan, and Australia); North America (Canada and USA); Northern Europe (Denmark, Finland, Iceland, Norway, and Sweden), and Western Europe (France, Spain and Great Britain). The study observed an increase in the prevalence of drug use in children and adults since 2000 in all regions, varying across regions and countries studied. Assuming that the prevalence of ADHD is similar worldwide when consistent diagnostic criteria and methods are used, the authors suggest that the variation in the use of medication may be related to: the different ways in which the diagnostic criteria are applied in practice; the structure, functioning and financing of health systems (for example, direct access to specialists, availability and cost of medication and availability of non-pharmacological treatments); proportion of off-label drug use and cultural variations in perception and treatment of ADHD. Moreover, such variation may still be associated with the influence on doctors of the guidelines and studies validating the effectiveness and/or safety of medications, as well as the long-term consequences of inappropriate treatment. While recognizing that there is no clear evidence on optimal rates of prescription, the study results indicate that many patients are likely to be treated inappropriately (especially in areas of low drug use), whereas others may be overtreated (Raman et al., 2018).

Raman et al. (2018) do not display data from Latin America, which indicates a significant information gap and exposes an important challenge regarding the consolidation of useful data not only for decision making and policy strategies, but also for the production of knowledge about the condition in LMIC. Furthermore, as noted below, it does not reflect the intense debate in about etiology, diagnosis, treatments and organization of care for patients living with ADHD.

GMH interventions for ADHD are therefore important to overcome the diagnosis and treatment gaps. Still, one should be wary of Western psychiatric classification systems and care models with little scalability outside HIC. The privilege of biomedical models of diagnosis and treatments frequently disregards local historical and sociocultural contexts. Issues of overdiagnosis, false positives, inadequate therapy and adaptation to local contexts are seldom or insufficient addressed.

Many of the arguments already presented by advocates and critics of the GMH agenda for ADHD are taken up in a particular form in the debates around the condition in Brazil, which we will now expose, after presenting data on ADHD prevalence and on the production, prescription and consumption of methylphenidate in the country.

## The Biosocial Field of ADHD in Brazil

### Epidemiology of ADHD in Brazil

As in other countries, the prevalence of ADHD in Brazil evinces a huge variation among different studies. The Brazilian sanitation regulatory agency (ANVISA) estimates that the prevalence of the condition ranges widely, from 0.9 to 26.8% (Boletim Brasileiro de Avaliação de Tecnologias em Saúde (BRATS), 2014).

Several epidemiological studies in the country found 3.6 to 5% prevalence among school-age children (Barbosa and Gouveia, 1993) and other study estimates the prevalence of 3–6% in children aged 10 to 14 (Oliveira et al., 2016). Rohde et al. (1999) found a prevalence of 5.8% in a sample of teenagers based on DSM-IV criteria. Among students at four public schools in the state of Rio de Janeiro, Fontana et al. (2007) found a prevalence of 13%; yet in the same region of the country, a different study showed a prevalence of 17.1% (Vasconcelos et al., 2003). It is important to highlight that higher prevalence has been found in socioeconomically disadvantaged populations, which usually attend public schools in Brazil (Oliveira et al., 2016). Freire and Pondé (2005) studied 763 children attending a public school in Salvador, in the state of Bahia in northeastern Brazil. They estimated that 12 children (8%) had a high probability of having ADHD. Another study conducted in a different public school in Salvador in 2016 with 265 children ages 10 to 17 showed prevalence of 16, 6% (Oliveira et al., 2016). Guardiola et al. (2000) found 18% prevalence based on DSM-IV criteria in a sample of 35,521 students ages 6–14 in elementary schools in the city of Porto Alegre, in South Brazil. Of those students, 64.7% were in state public schools, 11.9% in municipal schools, and 23.4% in private schools. It has been highlighted that studies which use DSM-IV criteria have shown a higher prevalence than those using previous versions of DSM (Baumgaertel et al., 1995).

Thus, there is a huge variation in prevalence of ADHD across the country. As in other national contexts these discrepancies have fueled arguments of those who argue for the “social construction” of the condition driven by the interest of the pharma industry. Against this backdrop a widely cited meta-analysis of the global prevalence of ADHD conducted by Brazilian researchers linked the differences in prevalence to the diversity of methodologies used in the different studies. Taking this factor into account they estimated a global prevalence of ADHD of 5% among school-age children (Polanczyk et al., 2007). Other Brazilian scholars associated the variations in prevalence to the type of sample examined, the different instruments and diagnostic criteria and then observed a significant difference according to the person providing the information, whether the parents, teachers, or the children themselves (Rohde et al., 1998; Vasconcelos et al., 2003). Debates and controversies around ADHD in the country draw on the variations in the prevalence either to argue for over–or underdiagnosis of the condition.

### Production, Prescription and Consumption of Methylphenidate in Brazil

The global production of methylphenidate increased from 28.830 kg in 2001 to 70.669 kg in 2017 (International Narcotic Control Board (INCB), 2018). Around 268 kg of methylphenidate were sold in Brazil in 2005, reaching 875 kg in 2012 (Barros, 2014). More recent data is unavailable, since Brazil and other major producers did not report their data to the International Narcotics Control Board (International Narcotic Control Board (INCB), 2013). In 2017 the country ranked seventh among the major importers of the medication (International Narcotic Control Board (INCB), 2018).

Since the early 2000's, reports about the pharmaco-epidemiological distribution of controlled substances such as amphetamines and other appetite inhibitors and methylphenidate have been one of ANVISA's priority targets. Regarding the consumption of methylphenidate, the country had no electronic system for regulating controlled medications before 2007 (Relatório 2009–Sistema Nacional de Gerenciamento de Produtos Controlados (SNGPC), 2010). Local inspectors had to check records of purchases and sales by drugstores. ANVISA did not have access to these local data (Noto et al., 2002). According to the regulatory agency, the consumption of methylphenidate in Brazil in 2009 ranged from 5 kg in January to 20 kg in October (the discrepancy is attributed to the consumption decrease during school holidays). Data from the new information system reported an annual consumption of 156.623 kg in 2009, 266.092 kg in 2010, and 413.383 kg in 2011 (Sistema Nacional de Gestão de Prescrição Controlada (SNGPC), 2012; Gomes et al., 2019). Data presented by the Ministry of Health in 2015 declared that Brazil has become the second world market in methylphenidate consumption, with about 2,000,000 boxes sold in 2010, and indicated a consumption increase of 775% in the last 10 years in the country (CONANDA, 2015).

Methylphenidate demands a specific prescription for narcotics and psychotropic medication. The physician has to register at the corresponding regional council of medicine (CRM) to get the prescription for those substances. This requirement has faced strong opposition from many Brazilian physicians, who disagree with the current methylphenidate regulations. They argue that the procedures are excessive and outweigh the potential risks. Moreover, the notification process may intimidate and embarrass patients and multiply unnecessary bureaucratic procedures (Carlini et al., 2003).

In Brazil, the Ministry of Health does not include methylphenidate in its standardized dispensing lists via the Unified Health System (SUS), such as the National List of Essential Medicines (RENAME). Nevertheless, States and Municipalities have relative autonomy to include specific medications according to their local particularities. Parent and professional associations have exerted pressure to include methylphenidate in the lists cited above. Hence, under certain criteria SUS patients in the state of Espirito Santo and the cities of Sao Paulo and Campinas, in South East Brazil, have access to the medication.

The state of Espirito Santo included methylphenidate in its List of Essential Medicines (RENAME) (Espírito Santo, 2007) and was the first state in the country to create an ordinance regulating the public dispensation of the medication in September 2010 (Caliman and Domitrovic, 2013). It was followed by the municipality of São Paulo, in June 2014 (São Paulo, 2014) and by the city of Campinas, in October 2014 (Campinas, 2014). However, the three regulations diverge regarding inclusion criteria, such as age and symptoms, the dispensation place and the professional who may prescribe the medication. In Rio de Janeiro, a municipal act was approved but it did not suffice to establish a program for public dispensation (Rio de Janeiro, 2012). In 2012, former mayor Eduardo Paes approved draft act n. 710/2010, which granted rights to students with ADHD in the city of Rio de Janeiro. It established guidelines for parents and teachers and also determined the availability of medicines in municipal public health facilities (Esher and Coutinho, 2017).

The relative autonomy of each state and municipality to define their own list of “essential medications” ensures that some medications, which until then could only be accessed by the population through lawsuits against the state, may be requested through administrative processes in SUS state pharmacies (Caliman and Domitrovic, 2013). Critics underline the lack of national policies for ADHD treatments and the complexity involved in granting access for procedures for low-income patients to obtain methylphenidate within SUS (by lawsuits or through an administrative process beset by red tape). Maia et al. (2015) estimate that only the direct consequences of not treating children with ADHD ages 5–19 would amount ~R$ 1,841 billion/year (Brazilian real), and if the country increases its investment in treatments from the current R$ 28 million spent by families out of pocket to R$ 377 million, the amount saved would be 3.1 times higher than the expenses.

### Debates and Controversies Around ADHD in Brazil

#### Background: The Brazilian Psychiatric Reform and the Organization of Mental Health Services

The Brazilian mental health system has ensued from the psychiatric reform, which ran parallel and partly overlapped with the Brazilian health care reform (Fleury, 2011). The latter resulted from the Constitution of 1988, enacted after the redemocratization of the country following the end of the dictatorship and which led to the creation of the Brazilian Unified Health System (Sistema Único de Saúde–SUS, Paim et al., 2011). The SUS system is organized regionally, with a decentralized network of health services formed by a complex set of public, private and philanthropic providers, under coordinated management at each level of government and with strong community participation (Lobato and Burlandy, 2000; Souza, 2002). The regionalized structure of SUS starts at the municipal level with relative autonomy to define its own health policies and structures of care. This will have important consequences for the field of ADHD in the country, as we will show.

Since the establishment of the SUS there have been continuous efforts toward universal health coverage. Regrettably, the Constitution also enabled the participation of private health insurance companies in a “complementary” way to the public system. Hence, on a constitutional and legal basis, Brazil has a universal public health system, though in practice, health financing is mostly private due to the underfinancing of the public system and the absence of clear limits to the participation of private health companies (Oliveira and Dallari, 2016).

The Brazilian psychiatric reform was inspired by the Italian reform and the Italian democratic-psychiatry movement led by Franco Basaglia in Trieste. An additional influence was the Lacanian-inspired institutional psychotherapy program of La Borde in France (Barros, 1994; Passos, 2009; Foot, 2015). In the early 1970's, Brazilian mental health workers initiated the so-called “anti-asylum struggle” (luta antimanicomial) and criticized the psychiatry establishment's collaboration with the dictatorship. This movement developed within the context of the country's democratization and the sociopolitical mobilization of that time (Delgado, 1992; Amarante, 1995; Tenório, 2002). Guidelines proposed by the Pan American Health Organization (1990) were adopted resulting in the reorientation of mental health care from a hospital-centered system to community mental health care and primary health care models. Mental health policies in the country were consolidated in 2001, when the Psychiatric Reform Law (Federal Law 10.216) was approved, which grants the protection and rights of those with mental disorders and redirects the model of care in mental health (Csillag, 2001). It encourages the programmed reduction of long-term admissions in psychiatric wards, and whenever possible the replaced replacement by beds in general hospitals for short periods of time. It also promotes the deinstitutionalization of psychiatric inmates and their psychosocial insertion through work, culture and leisure (Ministério da Saúde, 2004).

The mental health law contemplated the development of Psychosocial Care Centers (Centros de Atenção Psicossocial–CAPS), which is considered the keystone of the Brazilian Psychiatric Reform. CAPS are community mental health services that provide outpatient care or partial hospitalization for patients with severe mental illness, and are articulated with primary care units to organize psychiatric care in a defined catchment area, defined as “territory” (Mateus et al., 2008). Based on the universal logic of public health care CAPS system opposes framing mental health policies and specialized services according to specific diagnoses. Hence, the notion of a “citizen burdened by mental suffering” should replace different psychiatric labels and diagnoses (Ministerio da Saúde, 2004; Biehl, 2005: p. 134). Diagnoses at CAPS and CAPSi (specific form of CAPS for children and adolescents) are always ongoing processes and not definitive, but re-assessed according to the corresponding “care strategy,” which may involve psychotherapy, rehabilitation, group activities and medication. At the center of the process is the singularity of the child–i.e., the child's history, family and everyday life (Couto, 2004, 2012; Couto et al., 2008)–and the “primacy of the ethical” to promote the patient's unique experiences and his position as a moral subject (Goldberg, 1994; Biehl, 2005). Thus, the so-called projeto terapeutico singular (unique therapeutic project) is the main tool for the work conducted at CAPS and reflects the strong imprint of psychoanalysis and its emphasis of singularity (Cunha, 2007). These strategies embody the basic tenets of the so-called Psychosocial Care Paradigm, considered an epistemological turn in mental health (Yasui et al., 2016). Correspondingly, human suffering is addressed in its complexity, as part of a psychosocial dynamics that suspends the labels of normality and sanity and examines the social and biopolitical interests and mechanisms behind those labels. Care is no longer understood as therapeutic isolation or moral treatment, but as “creation of socialities and subjectivities” and the patient is “no longer an object of knowledge, but a subject expressing insanity” (Yasui et al., 2016: p. 401).

Several parent associations oppose CAPSi principle of not organizing services according to specific diagnoses, demand specialized services for their children along with the involvement of the associations as political actors and criticize the limited connection to other sectors, such as education and social assistance (Nunes, 2016). Moreover, they also strongly disagree with psychoanalytic treatments at CAPSi. Despite claims to multidisciplinarity, several CAPSi–particularly those in the State of Rio de Janeiro, have a psychoanalytic orientation and promote a discourse of self-sufficiency and exclusivity of psychoanalytic treatments (Lima et al., 2018).

Professionals working at CAPSi do not consider ADHD as a severe condition and many are harsh critics of the disorder. Additionally, they resisted the introduction of specialized services and advance, rather demedicalizing strategies for issues of hyperactivity and attention problems. Conversely, parent and psychiatric associations advanced a biomedical understanding of the condition and have criticized the lack of specialized services for children with ADHD. There are very few specialized services for ADHD within SUS or the private sector^1^. Controversies around ADHD in the country have gravitated around the issue of medicalization of social problems, involving other medical and non-medical actors. Such debates have strongly impacted policy orientations, mental health care setup, professional knowledge and care practices, as we will further examine.

#### ADHD Activism

Debates around ADHD in the country oppose groups that support biomedical descriptions and classifications based on the countless scientific studies published on the topic and those that challenge biomedical models and psychopharmacological treatments (Associação Brasileira de Saúde Mental (ABRASME), 2010; Fórum sobre Medicalização da Educação e Sociedade, 2011; Mattos et al., 2012; Associação Brasileira de Psiquiatria (ABP), 2014). While the former advances a biomedical discourse and incorporates arguments from the GMH agenda, which stresses the treatment gap for the condition in Brazil, the latter draws on the medicalization narrative.

Different from countries like the US, Canada, UK or Australia, Brazil does not have such a strong tradition of patient support groups. Still, there are a growing number of associations for patients with autism, ADHD, and obsessive-compulsive disorder and their family members. These associations play an important role in disseminating knowledge about these conditions and fighting for treatments, services and citizens' rights (Frossard, 2015; Rios and Andrada, 2015, 2016; Nunes and Ortega, 2016).

Parallel to the development of patient and parent associations for ADHD, autism and other conditions in Brazil, and in the context of the psychiatric reform along with the following deinstitutionalization process, there have also emerged a small number of users' and survivors' groups. They are very few, and not restricted to service users, encompassing family members (mostly from lower classes) and professionals from public mental health services. Their role has been rather marginal when compared to the influence of those groups in other countries due to the lack of structure to organize their activities as well as insufficient funding and meager political support (Vasconcelos, 2013; Almeida, 2019). Several factors account for this precarious situation, including, the strong hegemony of patrimonial and hierarchical culture within Brazilian society resulting in a vertical organization with professionals on top of the hierarchy, enormous social inequality and the limited presence of public initiatives for mental health within the neoliberal public health policies (Vasconcelos, 2013). These organizations gravitate around the CAPS system and therefore promote the universal logic of public health care and oppose specialized policies and services for separate conditions.

Likewise, many CAPSi do not keep relations with family associations, in disagreement with the ideal of collaboration implicit in the principles of the psychiatric reform (Lima et al., 2017, 2018). This lack of dialogue is one of the reasons for the dissatisfaction of parent associations with mental health policies and services in the country. In Brazil, the middle class does not traditionally frequent public mental health services and users from lower classes have greater difficulties and economic, social and cultural limitations to engage in political activism (Vasconcelos, 2013). Thus, potential users and family members with greater educational, social and economic resources who may expand and give visibility to psychosocial care related activism, are not involved in such forms of activism. These individuals have opted for associations promoting biomedical treatments and specialized services for specific conditions, such as ADHD or autism (Vasconcelos, 2013).

Most ADHD associations in Brazil include patients, relatives, professionals, and researchers. The most important group with the highest level of visibility, political articulation and regional influence is the *Associação Brasileira do Déficit de Atenção* (ABDA). It is a non-profit organization created in 1999 with the aim of disseminating knowledge about the condition. Located in Rio de Janeiro, ABDA does not have local chapters but it has local support groups involving professionals, family and patients in the main cities of the country. With circa 200,000 visits a month its website (www.tdah.org.br) is a powerful vehicle of information; and the mixed membership of ABDA enables the dialogue between professionals and lay public and the spreading of a biomedical discourse around ADHD in the country. ABDA has a unique connection with pharmaceutical companies. Novartis and Shire Pharmaceuticals are key sponsors of the association. The mission of ABDA is to convey a biological discourse on ADHD, its causes, diagnosis and treatments. ABDA does not provide clinical assistance, diagnostics or any type of treatment. ABDA is focused on advocacy, information about professionals and specialized services and the dissemination of knowledge (https://tdah.org.br/a-abda/quem-somos/). The ABDA website is likewise used by professionals and researchers to comment on articles on ADHD in the mainstream press, and to share their views on the validity or legitimacy of such articles (Ortega et al., 2018). There are also smaller associations, such as Inspirare (http://www.associacaoinspirare.com.br/), which focus on ADHD, autism and dyslexia. It is a non-profit organization based in Sao Paulo and its aim is disseminating knowledge about the conditions, providing support groups and information about health and educational policies, as well as advancing advocacy and the fight for social and educational inclusion. No connections with pharmaceutical companies are known for Inspirare.

The *Grupo de Estudos do Déficit de Atenção* (GEDA), a research group associated with the Institute of Psychiatry of the Federal University of Rio de Janeiro, and the *Programa de Transtornos de Déficit de Atenção/Hiperatividade* (ProDAH; www.ufrgs.br/prodah) belonging to the Faculty of Medicine of the Federal University of Rio Grande do Sul, in Southern Brazil, have important links with ABDA. Some of their members are also on the board of ABDA. These research groups respond for most of the research on methylphenidate and ADHD published in the country. They also receive substantial funding from drug companies. ABDA and the associated research groups are engaged in producing research on ADHD, promoting patient and family support groups, and are the main drivers of the official biomedical discourse about the disorder in the country (Ortega et al., 2018).

The main scientific journals in Brazil, such as the *Jornal Brasileiro de Psiquiatria* mostly publish research by those groups, frequently with advertising from Concerta. Authors frequently disclose their funders, which in several cases are not explicitly declared. As an illustration, a supplement of the *Jornal Brasileiro de Psiquiatria* on ADHD, which was published in 2007, was funded entirely—from the articles to the advertisements—by a manufacturer of methylphenidate (Jornal, 2007). A study that compares scientific publications and newspaper and magazine articles in Brazil found that while scientific publications stressed the use of methylphenidate for ADHD treatment, lay publications included other approaches and also mentioned social pressures and demands as factors that influence individual attention and concentration (Itaborahy and Ortega, 2013). Moreover, the study also found that scientific and lay articles diverge on the role attributed to psychotherapy in the treatment of the disorder. While the former that the combined use of the medication and psychological therapies yielded worse results than the use of the drug alone, the latter emphasized the benefits of psychotherapy in treating the condition (Itaborahy and Ortega, 2013). This is also significant in a country where psychoanalysis is an important theoretical and clinical perspective in public mental health services, and the issue of its “scientificity” and evidence-based standards has come to close scrutiny and to inflamed controversies. Together with critical and anti-psychiatric models, psychoanalysis contributes to challenge biomedical understandings of ADHD that consider behavioral problems as symptoms of underlying pathological conditions (Hinshaw et al., 2011; Conrad and Bergey, 2014).

Parent and professional associations have embraced the disability model and joined demands for specialized services and policies for ADHD. However, ADHD associations did not succeed in cementing a disability perspective. Pressure to understand ADHD as disability challenges the universal logic that structures the model of public health in the country as a vehicle for the rights of those individuals to treatments, specialized services through the Health Care to the Person with disability Network and social and educational inclusion.

This approach is in line with the initiatives of several professionals who have sought to reinvigorate their demands and political articulation, occupying relevant spaces within the media, parliament, judicial system and health managers (Vasconcelos, 2012). Those professionals have approached patients and family members through organizations such as ABDA, thereby not only legitimating biomedical discourse and interventions for the condition but also reproducing the patronizing logic of the associations in which professionals occupy the most prominent role vis-a-vis patients and family members.

The alliance between families, health and education professionals, policy makers, and the pharma industry in the country has built a successful lobby to propose bills and municipal, state and federal laws associated with ADHD and dyslexia over the last 10–15 years. Most bills and laws concern the school system, including the “diagnosis and treatment in the public basic education system” (Senate Bill 3517/2019), even determining the location of classroom chairs for children with ADHD in public and private schools in the state of Rio de Janeiro (Law 8192/18), and promoting free supply of medication and awareness campaigns (Lima, 2019).

As we already mentioned in the first sections of the article, GMH is currently one of the most powerful narratives for advancing ADHD specific policies, services and treatments and, what is more important for patients and family associations, the sponsor of advocacy groups (Flisher et al., 2010) The GMH agenda is primarily intended for low-income countries and Brazil is a middle-income country with a well-organized National Health System (SUS). However, GMH discourse, methods and metrics, such as the emphasis on the global burden of disease of ADHD and the disability-adjusted life years—DALYs lost to the condition, as well as the development of evidence-based interventions and treatments, have been taken up by health professionals and family associations not only to advance biomedical and behavioral approaches to ADHD but also, as a basis to criticize public mental health policies, services and interventions in the country. Specifically, those actors strategically draw on the GMH rhetoric and methods to justify and legitimize a certain view on diagnosis, treatments and service organization and to criticize the hegemony of psychoanalysis in public mental health services because it lacks “scientificity.”

#### Treatment Gap and Accessibility

Global Mental Health and WHO strongly promote the concept of “treatment gap” which refers to the difference that exists between the number of people who need care and do not receive it, or receive it inadequately and those who receive it (Jacob et al., 2007; Patel et al., 2007; Saxena et al., 2007). This view emphasizes not only personal suffering, but also disability and economic losses for individuals and countries due to non-treatment (Jacob et al., 2007; Patel et al., 2007; Saxena et al., 2007). The estimate of the treatment gap depends on the prevalence of the disorder, the period of analysis of service use and the representativeness of the sample in relation to the population under analysis (Kohn et al., 2004). Therefore, its calculation derives from the adoption of standardized diagnostic criteria and epidemiological estimates of prevalence and information about monitoring by health services (Kohn et al., 2004). In this sense, it reveals both the lack of treatment and the lack of information in countries with less resources (Jacob et al., 2007). On the other hand, the treatment gap logic hides the diverse arrangements that take place in different contexts involving actors, institutions, knowledge and therapeutic practices (Bartlett et al., 2014; Orr and Bindi, 2017). Furthermore, as it bases its findings on psychiatric categories and epidemiological data, it can foster reductionist biomedical approaches (Jansen et al., 2015).

The diverse Brazilian groups promoting biomedical models, specialized services and specific health and education policies for ADHD share the global mental health discourse emphasizing a treatment gap for the condition. Thus, in an effort to support the hypothesis that ADHD is undertreated and underdiagnosed in Brazil, the researchers Paulo Mattos et al. (2012) published a letter to the editors of the official journal of the *Associação Brasileira de Psiquiatria* (www.abp.org.br) in which they argue that even if lower prevalence estimates were used instead of the expected prevalence, it could still be concluded that more than 80% of individuals with the disorder were not receiving medication-based-treatment (Mattos et al., 2012).

The Global Mental Health agenda combines discussions around treatment gap with a human rights discourse. Thus, to deny access to medication constitutes a human rights violation (Fernando, 2014). However, as we have seen, this is a very complicated issue. In a country like Brazil where there are strong controversies around issues of etiology, diagnosis, treatment, services and policies for a condition, whose very existence is being challenged, to advance biomedical discourses and treatments using an ethical and human rights alibi to coerce into its enforcement can further inflame an already polarized situation. Furthermore, it can hamper serious discussions and possible cooperation between mental health professionals and ADHD associations. Hence, we agree with Fernando (2014: p. 161), who claims that in the field of Global Mental Health the call on “human rights” should be restricted to “arguments for the rights of people to freedom, liberty and the exercise of personal choice,” and any pressure to “argue for alleged ‘rights’ to ‘treatment’ or even ‘care,’ referring to models of what these mean in the West” should be met with extreme caution.

Human rights of individuals living with mental disorders are also a central concern for Brazilian public mental health, and are thematized in a broader perspective when compared with the limited understanding frequently associated with global mental health. Since the emergence of the psychiatric reform movement during the military dictatorship serious violations of human rights of imamates of psychiatric hospitals have been systematically denounced. The issue of guaranteeing human rights for people with mental disorders have expanded in the following decades for it was not a simple matter to humanize psychiatric hospitals, but rather to positively affirm the patients' right to fully exercise citizenship and to build community-based, intersectoral care strategies that advance social inclusion (Delgado, 2011; Amarante and Nunes, 2018; Almeida, 2019). The issue of access and right to treatment is part of the human rights agenda, as well as the approval of legislation to regulate interventions and services, the encouragement of social participation, and the awareness of society to the challenges posed by mental illness. There is a prolific debate around access to healthcare in the country and it is not limited to the evaluation of metrics related to diagnosis and treatment according to biomedical standards (Menezes et al., 2018; Almeida, 2019).

Hence, Brazilian discussions of access and accessibility are more nuanced and broader than discussions around treatment gap within GMH (Travassos and Martins, 2004; Andrade and Minayo, 2012). Brazilian studies acknowledge the polysemic and multidimensional character of the issue of accessibility and its associated political, socioeconomic, organizational and symbolic aspects. Therefore, accessibility transcends the mere availability of health services (Andrade and Minayo, 2012; Assis and de Jesus, 2012). Moreover, the evaluation of the different determinants of accessibility involve a diversity of approaches (Assis and de Jesus, 2012). Several studies examine the issue of access to mental health care and are characterized by the multiplicity of methodologies and conceptual articulations. Subjects such as community care, social determinants of health, equity and human rights modulate the debate. Although Brazilian literature on mental health evinces a variety of studies and local perspectives on the issue of access, this body of knowledge is fragmented, making it difficult to systematize their findings (Menezes et al., 2018).

#### Critiques to Medicalization of ADHD

The biosocial field of ADHD in Brazil is highly polarized. On the one hand, parent associations and biomedical oriented professionals promote biomedical views, specialized services and special policies for ADHD, as well as claim that there is a treatment gap in the country and the condition is underdiagnosed. On the other hand, and as reaction to those groups several public directives, recommendations and protocols have regulated prescriptions of methylphenidate and challenged biomedical diagnosis and treatments of ADHD in Brazil. The medicalization discourse is advanced by CAPSi professionals and other groups made up by education and health professionals who articulate with state councils of psychology and other political actors to promote those recommendations and protocols, as we will now examine.

A directive issued by the Secretaria Municipal de Saúde of São Paulo, the city's department of health, in 2014 regulates the prescription of methylphenidate and ADHD treatments and determines the referral of ADHD patients to CAPSi. This determination echoes the national mental health policy and states that “from a clinical perspective, it is a complex task to distinguish cases of ADHD from parts of educational problems which derive from inadequate educational models for the children's social context, increasingly complex family issues, and a sociocultural context in which there is competition, production of stigmas, and exclusion” (Portaria, 2014).

The *Associação Brasileira de Saúde Mental* (ABRASME) (www.abrasme.org.br), an association aligned with the psychiatric reform and the mental health policy in the country, supported the regulation because it assesses the complexity of the condition and its management (Associação Brasileira de Saúde Mental (ABRASME), 2010, 2014). On the opposite front, the directive of the *Associação Brasileira de Psiquiatria* (ABP), traditionally critical of the deinstitutionalization process and the mental health policy in the country, published an open letter [entitled “Carta Aberta a População” (“Letter to the People”)] challenging the São Paulo directive, arguing that the regulation “is restrictive, bureaucratizes respectful access to treatment, mainly by people at a social disadvantage, and positions itself against scientific systematization in a mystifying, disrespectful way.” In addition, it constitutes an “abusive barrier to access to pharmacological treatment by people with low income, and places restrictions on the full exercise and autonomy of Brazilian medicine and science” (Associação Brasileira de Psiquiatria (ABP), 2014; Ortega et al., 2018).

In the same year of the publication of the Sao Paulo directive, the Brazilian Bulletin on Health Technology Assessment (BRATS) issued a study that evinces the “high potential for abuse and dependence” of the medication and demanded “thorough assessment of the effect of methylphenidate on ADHD” (Boletim Brasileiro de Avaliação de Tecnologias em Saúde (BRATS), 2014). The authors highlighted methodological flows, short-term follow-up and low generalizability of the studies that addressed the efficacy and safety of methylphenidate among children and adolescents. They also stressed the number of false positives and unnecessarily treated children, given that several symptoms are also found in individuals without ADHD (Boletim Brasileiro de Avaliação de Tecnologias em Saúde (BRATS), 2014; Esher and Coutinho, 2017). Machado et al. (2015) strongly opposed the study arguing that such conclusions could not be inferred from the studies cited in the BRATS document.

Further directives and recommendations have been issued in recent years. Inspired in the Sao Paulo directive and a similar protocol in Campinas, the National Health Council released a public document entitled “Recommendations of the Ministry of Health for the adoption of non-medicalizing practices and for the publication of municipal and state methylphenidate dispensing protocols to prevent excessive medicalization of children and adolescents,” and following the publication of data asserting that Brazil has become the second world market in methylphenidate consumption, the National Council for the Rights of Children and Adolescents (CONANDA) issued the Resolution 177/2015 which “provides for the right of children and adolescents not to be subjected to excessive medicalization, especially regarding learning, behavior and discipline issues” (CONANDA, 2015). The Ministry of Health also issued a recommendation for states and municipalities to publish methylphenidate dispensing protocols “to prevent excessive medicalization of children and adolescents^2^”.

These documents and the professionals and organizations that support them share the concern with the “excessive medicalization” and promote the adoption of “non-medicalizing practices.” They challenge limited understanding of access and accessibility to treatments and health services associated with treatment gap arguments and advocate for a broader and more complex understanding of the issue of accessibility. Hence the medicalization narrative shares some of the arguments prevailing in discussions around broader understandings of accessibility. While the former tends to adopt a more radical and polarizing perspective opposing ADHD policies and treatments the latter is more nuanced and favors an attentiveness to the specific contexts and their differences regarding access to treatments and services.

The medicalization discourse is championed not only by many mental health professionals working at CAPSi and other public facilities, but also by groups such as *Fórum sobre Medicalização da Educação e da Sociedade*, whose founder the pediatrician Maria Aparecida Moyses participated to the São Paulo directive regulating the prescription of methylphenidate in the city. The forum was created in 2010 as a reaction to programs for dyslexia and ADHD in the city of Sao Paulo. The forum argued that the programs addressed social and educational problems from a biomedical perspective. From the beginning, the forum that gathers professionals from diverse areas, especially health and education, and involves state councils for psychology has established collaborations with different groups in Brazil and other countries (Fórum sobre Medicalização da Educação e Sociedade, 2013). As a result, after a meeting with an Argentinian movement, Forumadd (www.forumadd.com.ar), which also opposed the pathologization and medicalization of childhood, a document was issued establishing the collaboration and principles of the movement with the goal of spreading awareness among diverse groups and associations through Latin America. Their strong stance against medicalization involves opposing the increasing consumption of methylphenidate and other psychotropics among children and adolescents arguing that learning difficulties and ADHD are not pathologies, or simply “do not exist,” and challenging policies to address those conditions (Fórum sobre Medicalização da Educação e Sociedade, 2011; Ortega et al., 2018).

In 2014 the Movement *Despatologiza—Movimento pela despatologização da vida* (https://www.despatologiza.com.br) was found by professionals across different fields, such as medicine, education, psychology, speech therapy as well as researchers from those fields and service users with the goal of collectively start “facing pathologization processes that transfigure differences in diseases to hide the inequalities that plague our society.” The processes of pathologization of life, they argue, are displayed in “exaggerated or even mistaken diagnoses and interventions,” by which the pathologization of children and adults expands both in scientific and lay discourses across practices and services in all areas, from education to mental health. The movement is really active and gathers professionals, researchers and users from different regions of the country. The “suggestions of depathologizing practices” encompass actions like continuous training for managers and coordinators of the Public Education Network to encourage depathologizing actions involving educators; the continuously monitoring of Legislative Bills to avoid the approval of laws of dyslexia and ADHD and, more broadly, to stimulate more depathologizing practices in health, education and social assistance areas.

Medical and non-medical actors (family associations) have relied on Global Mental Health discourses and its metrics to substantiate the scientificity and evidence-based of biomedical and behavioral approaches to ADHD and to claim for bridging the treatment gap. The existence of ADHD as a discrete entity is still disputed. Its ontological status and boundaries are hotly debated. For those who opposed biomedical approaches ADHD constitutes an illustration of medicalization of attention, understood as a process by which non-medical problems come to be defined and treated as medical problems, either as diseases or disorders (Conrad and Bergey, 2014; Zorzanelli et al., 2014).

On the other hand, as recent studies have shown, there has also been an expressive increase in the use of methylphenidate in Brazil unrelated to the diagnosis of ADHD. Such studies have observed that the growth is associated with the fact that prescriptions are largely done in the private sector (for middle- and upper classes) and to the off-label use for cognitive, physical, sexual and emotional enhancement also related to those classes (Coutinho et al., 2017; Esher and Coutinho, 2017; Lima et al., 2019; Castro, 2020).

The use of medication unrelated to medical diagnoses has been described as “pharmaceuticalization” and aims to enhance performance. There is still not enough data to understand the extent and impact of methylphenidate pharmaceuticalization in the country, but several scholars insist that it is a social and ethical problem that requires further investigation and action by the regulatory agencies of drug dispensation (Coutinho et al., 2017; Esher and Coutinho, 2017; Lima et al., 2019; Castro, 2020). Both phenomena, medicalization of ADHD and off-label use of methylphenidate, highlight the challenges imposed by the globalization of ADHD and the importance of ethically informed and ecologically sensitive clinical practices (Singh et al., 2013).

The increase of prescriptions in the private sector and the off-label use of methylphenidate for cognitive enhancement explains why despite methylphenidate is not included in the standardized dispensing lists of SUS, Brazil became, as already mentioned, the second world market in methylphenidate consumption. It also explains why there are still heated debates about lack of access to health care or even a “treatment gap. This offers a more nuanced view of the Brazilian situation which helps to move beyond polarized debates around treatment gap vs. medicalization. The difficulties in accessing methylphenidate in public health services heavily contrast with the huge dispensation in the private sector, leading to an uneven distribution of the medication according to social class and financing resources. As a result, part of the Brazilian population may be overmedicated, and critiques of medicalization of ADHD are well-placed and are relevant, while on the other hand, there is a problem of access not only to medication but also to social and educational inclusion for the poorer and vulnerable parts of the population. Therefore, those who claim that the condition is underdiagnosed and undertreated in the country are also right (beyond discussions of whether interventions and treatments should be restricted to psychopharmaceuticals or should also include psychosocial and educational interventions).

In contexts of extreme poverty and social vulnerability, pharmaceutical treatments are not foregrounded. As an illustration, an ethnography conducted in Nova Iguaçu, one of the poorest municipalities of the Rio de Janeiro Metropolitan Region, evinces that the lack of centrality of pharmacological treatments is associated to the fact that methylphenidate is not in the list of the essential medicines of the municipality (and therefore of free dispensation), and families with scarce resources cannot afford to buy it. The second reason is that, in those contexts, professionals emphasized social aspects of ADHD, such as violence, poverty, lack of parental authority, and of leisure activities (Chagas, 2017). Those families accepted the existence of the condition but they were fully aware of the impact of family and community relationships in the severity of the symptoms. The child's improvement was equally associated with contextual factors. Medication was not only regarded as a “luxury” item but as one that would not target the genesis of the problem (Chagas, 2017: p. 123).

## Discussion

In this article we have argued that the Brazilian case brings interesting elements to Global Mental Health discussions around ADHD. The local context is permeated by tensions that go beyond GMH research and interventions (Clark, 2014; Smith, 2017).

Brazilian scholars aligned with the notion of treatment gap have produced quantitative studies in which the contextual aspects that modulate the condition and search for treatments are not included. They seem to assume that ADHD is a natural entity engaging in a close dispute with professionals affiliated with the tradition of the Brazilian psychiatric reform, critical to the biomedical model (Ortega et al., 2018). Both actors have invested in academic production and political activism to advance their goals. Moreover, they have grounded their proposals on the notion of treatment gap. This notion is advanced to justify the need for ADHD research, interventions and funding. Moreover, it allows Brazilian data to be included in global studies. Nevertheless, Brazil has not yet produced enough prevalence studies of ADHD that reflect the national and regional situation and their differences. Such research requires funding and coordination that are not easily available in the national territory (Menezes et al., 2018). Besides, the notion of treatment gap, unlike that of access (Assis and de Jesus, 2012), does not have enough scope to explain the social conditions that produce the gap, in addition to the risk of overestimating the data because they are based on estimates produced from biomedical standards, such as instruments for detection and diagnosis and classification systems (Jansen et al., 2015).

Furthermore, there is an important question raised by Orr and Bindi (2017) of whether the treatment gap emphasized by GMH actually refers to an absence of treatment, or whether it is due to the lack of recognition of other modes of care because they are not evidence-based. In this sense, it is important to remember that contemporary societies, whether high-income or low- and middle-income countries, are characterized by the provision of multiple care practices, institutionalized or not, and it is therefore essential that research, interventions and public health policies consider local contexts and their medical traditions (Orr and Bindi, 2017).

Regarding the association of these research groups with family members and activists, what is observed is the induction of a demand for a specific type of treatment based on evidence produced in developed countries and focused on medication. This demand does not provide elements for patients and family members to think about diagnostic and therapeutic alternatives or structural mechanisms that may help in the provision of care (Ortega et al., 2018).

On the other pole of the debate, groups concerned with the medicalization of childhood are mainly associated with the field of public mental health (traditionally opposed to the Brazilian psychiatric association and family associations). In general, their academic production is related to the field of Collective Health (Vieira-da-Silva and Pinell, 2014), characterized by a wide variety of studies concerned with the different dimensions of the issue of access and with local populations and their concrete situations. Unlike other LMICs, Brazil has a health system that seeks to reconcile services and network assistance with scientific production. However, such studies are still poorly articulated with each other and do not allow for a perspective of the broader national context (Menezes et al., 2018).

Activism within public mental health services, although important for the Brazilian psychiatric reform, is not as strong as in several countries in the Global North. Furthermore, as we have already mentioned, it evolved with tensions and the distancing of professionals from users and their families. The fragility of the movements and the detachment from health professionals restrict their critical capacity and ability of promoting public policies and care for ADHD (Vasconcelos, 2013). While middle class associations of patients and family members have gradually been able to advance their agendas and extend their influence through national forums (Coutinho et al., 2017; Castro, 2020), associations gravitating around public mental health services struggle with advancing a broader perspective related to the logic of psychosocial care. Such imbalance in the imposition of the agendas of these different styles of activism reinforces the inequality in relation to access to treatments and health and educational services for children and their families. Moreover, it hampers the necessary discussion of comprehensive patient care and careful reflection about ADHD beyond the biology vs. biology/culture binary.

Another important aspect to understand the Brazilian context concerns the organization of the health system. Since the founding of SUS, it was understood that municipalization would be a strategy to strengthen local authorities' views in the decisions adopted by managers and, especially, municipal councils with the objective of increasing social participation and recognizing the particularities of each city. Such strategy would enable the adaptation of the necessary interventions for health promotion and assistance (Lobato and Burlandy, 2000; Souza, 2002). One of the consequences of this approach is that municipal authorities effectively configure access to treatments and follow-ups and, consequently, to medication directly, giving the debate a political character. City halls have relative autonomy to make decisions regarding the allocation of resources for health services (which includes dispensing medication), but health secretaries and mayors are under pressure from municipal health councils, a body made up of several representatives of a city's social movements that express their opinions and supervise the proposed measures.

The associations described in the previous section, such as ABDA and Inspirare are directly and indirectly related to this configuration, either when they are invited to participate in the development of consensus and guidelines with the SUS (municipal, state and federal) management spheres or when they associate with users and family members in initiatives such as those we described throughout this article. In this second case, what is observed is that such an alliance ends up generating greater demand for medication and even judicialization of the health system (Caliman and Domitrovic, 2013).

In this sense, the strategy of strengthening patient and family activism, also promoted by GMH, may induce the medicalization of ADHD. This is what Conrad and Bergey (2014) discuss regarding the factors that may be inducing the globalization of medicalization when they include the role of activism and the preference for DSM-based diagnostic criteria.

The Brazilian case is interesting for GMH because it enables the dialogue with experiences of health care, public policies and scientific production in mental health in a country characterized by diversity of mental health care services and strategies (Kieling and Belfer, 2012; Conrad and Bergey, 2014). The conflicting perspectives at stake update old controversies in psychiatry (such as the biological or cultural/social nature of mental conditions) and the challenges to improving research and intervention in mental health. Therefore, it is essential that GMH proposals are attentive to the production of research and interventions that articulate local and global knowledge, qualitative and quantitative methodologies, and, finally, but not least, biomedicine and social sciences. Otherwise, it puts itself at risk of becoming an agent that induces medicalization and the expansion of the Pharma industry (Clark, 2014; Mills, 2014).

## Conclusions

Historian Charles Rosenberg (2006) describes psychiatric categories such as ADHD as “problematic categories” with disputed boundaries and ontological status. ADHD, therefore, constitutes a “contested illness” (Brown et al., 2011), a condition exposed to public negotiations by medical and lay actors. We have seen in this article how the controversies around ADHD go beyond the medical system and encompass the public sphere and even popular culture, and involve professionals, parents, self-advocates, and social networks. Despite uncertainty about the etiology and the lack of convincing and well-replicated biomarkers with diagnostic or clinical utility (Visser and Jehan, 2009; Freedman and Honkasilta, 2017), ADHD is depicted by biomedical discourses as a neurobiological disorder, a brain disease. Furthermore, and in spite of the existence of GMH strategies for ADHD, there is no consensus regarding treatments and best forms of care. The call to bridge the treatment gap and to strengthen access to (primarily) pharmacological interventions is seen with suspicion by those who argue that in the absence of clear boundaries for the pathology, the lack of reliable biomarkers and the variation of prevalence across countries, regions and even cities, such a condition does not exist. Moreover, its main symptoms (attention deficit, hyperactivity, and impulsivity) fall within the range of behaviors expected in any given population (Ortega et al., 2018).

ADHD is at the center of controversies regarding its legitimacy and medical, social, epistemic, and ontological status. The category mobilizes legal arguments, administrative classification, and legislative maneuvers. Individuals diagnosed with ADHD, their families and professionals frequently become activists, mobilizing “scientific” or “moral” facts in favor of the condition's legitimacy and forming groups to share their experiences and fight for rights (Ortega et al., 2018). However, there is no “one” universal history of ADHD that depicts it as a universal disorder with clear boundaries and with similar prevalence across populations. What there are instead are local histories of ADHD in individual nations, and they reveal the condition as a “much more flexible, mutable phenomenon,” a notion that has been rejected as often as it has been accepted and that behavioral and educational problems “remain very much a product of local historical, cultural and political factors” (Smith, 2017: p. 786, 767).

In this article, we have told the national history of ADHD in Brazil against the backdrop of the global mental health agenda for the condition. Most of the arguments advanced by advocates and critics of GMH are taken up in a particular form specific to the history of ADHD in Brazil by those who claim that ADHD is underdiagnosed and undertreated, and that therefore there is an important treatment gap for the condition, and those who argue that the disorder is overdiagnosed and overtreated and speak of medicalization of childhood.

We have examined how global issues and controversies in mental health take local forms, illustrated in the issue of ADHD in Brazil. The national history of ADHD in the country is permeated by social, political, economic and cultural factors involved in the framing of diagnosis and treatments. Those factors are also present in other national histories of the condition. But they take up different configurations across nations. Examining the local and historical specificities alongside the practices of professionals make the abstract, globally circulating ideas meaningful in particular forms.

It is important that GMH research and interventions around ADHD are able to transcend and negotiate local and global knowledge production and practices and to integrate those binaries in the everyday life of subjects affected by the disorder (professionals, parents and individuals living with the condition). By considering such approach, global mental health may contribute to designing “better diagnoses” and reducing the threat of structural violence (Singh et al., 2013: p. 4, 5) in which the individualization of interventions can assume the restricted facet of increased access to drug interventions.

## Author Contributions

All authors listed have made a substantial, direct and intellectual contribution to the work, and approved it for publication.

## Conflict of Interest

The authors declare that the research was conducted in the absence of any commercial or financial relationships that could be construed as a potential conflict of interest.

## Footnotes

^1^https://tdah.org.br/category/profissionais-para-tratamento/locais-publicos-de-tratamento

^2^http://www.saude.gov.br/images/pdf/2015/outubro/01/Recomenda—-es-para-Prevenir-excessiva-Medicaliza—-o-de-Crian–a-e-Adolescentes.pdf
